# Evolution and Simulation of Terrestrial Ecosystem Carbon Storage and Sustainability Assessment in Karst Areas: A Case Study of Guizhou Province

**DOI:** 10.3390/ijerph192316219

**Published:** 2022-12-04

**Authors:** Tong Lin, Dafang Wu, Muzhuang Yang, Peifang Ma, Yanyan Liu, Feng Liu, Ziying Gan

**Affiliations:** School of Geography and Remote Sensing, Guangzhou University, Guangzhou 510006, China

**Keywords:** terrestrial ecosystem, carbon storage, land simulation, InVEST model, PLUS model, sustainable development, karst area

## Abstract

Against the background of “carbon neutrality” and sustainable development goals, it is of great significance to assess the carbon storage changes and sustainability of terrestrial ecosystems in order to maintain the coordinated sustainable development of regional ecological economies and the balance of terrestrial ecosystems. In this study, the terrestrial ecosystem carbon storage in Guizhou from 2010 to 2020 was assessed with the InVEST model. Using the PLUS model, the distribution of terrestrial ecosystem carbon storage by 2030 and 2050 was predicted. The current sustainable development level of the terrestrial ecosystem of Guizhou was evaluated after establishing an index system based on SDGs. The results showed the following: (1) From 2010 to 2020, the terrestrial ecosystem carbon storage decreased by 1106.68 × 10^4^ Mg. The area and carbon storage of the forest and farmland ecosystems decreased while the area and carbon storage of the grassland and settlement ecosystems increased. (2) Compared with 2020, the terrestrial ecosystem carbon storage will be reduced by 4091.43 × 10^4^ Mg by 2030. Compared with 2030, the terrestrial ecosystem carbon storage will continue to decrease by 3833.25 × 10^4^ Mg by 2050. (3) In 2020, the average score of the sustainable development of the terrestrial ecosystem was 0.4300. Zunyi City had the highest sustainable development score of 0.6255, and Anshun had the lowest sustainable development score of 0.3236. Overall, the sustainable development of the terrestrial ecosystem of Guizhou was found to be high in the north, low in the south, high in the east, and low in the west. The sustainable regional development of the terrestrial ecosystem of Guizhou was found to be unbalanced, and the carbon storage of the terrestrial ecosystem will keep decreasing in the future. In order to improve the sustainable development capacity of the terrestrial ecosystem, the government needs to take certain measures, such as returning farmland to forests and grasslands, curbing soil erosion, and actively supervising.

## 1. Introduction

A terrestrial ecosystem refers to the unity of terrestrial life and its environment on the Earth’s land surface. Carbon sequestration in terrestrial ecosystems is currently recognized as one of the most economically feasible and environmentally friendly ways to slow down the rise in atmospheric CO_2_ concentration by the international community [1]. Carbon storage in terrestrial ecosystems refers to the carbon stored in plant leaves, woody parts, and soil in the process of continuous carbon exchange among plants, soil, and the atmosphere [2]. Land use/land cover is an important factor that determines the carbon storage of a terrestrial ecosystem. When land cover changes from one type to another, it is often accompanied by a large amount of carbon exchange [3]. The impact of land-use activities on the carbon cycles of terrestrial ecosystems is far more extensive, far-reaching, and complex than industrial activities [4]. In 2020, during the 75th session of the United Nations General Assembly, China proposed scaling up its Intended Nationally Determined Contributions by adopting more vigorous policies and measures. China aims to have CO_2_ emissions peak before 2030 and achieve carbon neutrality before 2060 [5]. In 2015, 17 sustainable development goals (SDGs) and 169 specific targets were put forward by the Member States of the United Nations in a proposal to build a world where land degradation will no longer occur [6]. Against the background of the global emphasis on low-carbon sustainable development, it is conducive to understand the impact of land use on regional terrestrial ecosystem carbon storage under the action of human activities. Assessing the impact of terrestrial ecosystem changes on carbon stocks has become a necessary part of sustainable development.

The estimation methods of terrestrial ecosystem carbon storage mainly include the inventory method, vorticity correlation method, ecosystem process model simulation method, and atmospheric inversion method [7]. The inventory method can directly measure the carbon density of soil and vegetation, but it is a complex process that takes a long time. Additionally, there are differences between the carbon storage estimation of sample plots and whole regions [7]. With the wide application of GIS technology and various ecosystem models, as well as the gradual accumulation and enrichment of the carbon density results of sample plots in the early inventory process, research began to focus on the impact of land-use change on the carbon storage of terrestrial ecosystems. The DNDC (Denitrification-Decomposition) model [8], CASA (Computer-aided Sperm Analysis) model [9], CBMCFS3 (Carbon Budget Model of the Canadian Forest Sector) model [10] and Bookkeeping model [11] have been used to estimate regional land-use carbon storage. In addition to the above models, scholars are increasingly using the InVEST (Integrated Valuation of Ecosystem Services and Tradeoffs) model to calculate regional carbon storage [12,13,14,15,16]. The InVEST model has become a common model for estimating regional land-use carbon storage. Recent studies have explored the impact of land-use change [17,18,19,20,21] and socio-economic and population development [22] on terrestrial ecosystem carbon storage.

Large-scale studies at the regional scale consider areas containing urban agglomerations [23], provinces [21,24], and municipalities [25], and small-scale studies consider county [26], coastal zone [27], watershed [28], and arid areas [29]. Some of those studies used a land-use simulation model to predict the distribution of carbon storage given long-term land-use changes in the future. The common land-use simulation models include CLUE-S (Conversion of Land Use and its Effects at Small region extent Model) [30], CA (Cellular Automata)-Markov [31], FLUS (Future Land Use Simulation) [32] and PLUS (Patch-generating Land Use Simulation) [33,34]. For example, Mallick et al. estimated carbon storage and sequestration capabilities using the InVEST model and forecast the LULC map with cellular automata in Abha, Saudi Arabia, finding that the expansion of built-up land was the primary source of CO_2_ [35]. Li et al. simulated land-use patterns in Changchun, China, under three scenarios up to 2030 using the FLUS model and assessed carbon storage from 2010 to 2030 using the InVEST model [36].

However, few studies have focused on estimating and simulating the amount and distribution of carbon storage in karst areas. Karst areas have less flat land and more mountains. The terrestrial ecosystems in karst areas are fragile, with prominent ecological problems such as soil erosion and rocky desertification. Previous studies showed that soil carbon sequestration was higher in karst regions than in non-karst areas [37,38]. Therefore, understanding and simulating the changes in the carbon storage of terrestrial ecosystems are crucial for the future sustainable development and management of karst areas.

Guizhou is the largest karst landform province and national ecological civilization pilot area in China [38]. It has serious rocky desertification and soil erosion. Guizhou is also a province with significant forestry resources, large carbon storage in its forest ecosystem, and a strong carbon fixation capacity. In order to understand the relationship between the terrestrial ecosystem and carbon storage change and to explore ways to enact sustainable development in karst areas, the objectives of this study were as follows: (1) to analyze the distribution and characteristics of the terrestrial ecosystem carbon storage of Guizhou in 2010 and 2020; (2) to simulate future terrestrial ecosystem and carbon storage patterns in 2030 and 2050; (3) to scientifically quantify and evaluate the sustainable development capacity of the terrestrial ecosystem in karst areas based on SDGs. The results of this study are conducive to maintaining the balance of terrestrial ecosystems, promoting coordination between economic development and the ecological environment, and improving the long-term sustainable carbon fixation capacity of terrestrial ecosystems.

## 2. Materials and Methods

### 2.1. Research Area

**Figure 1:**
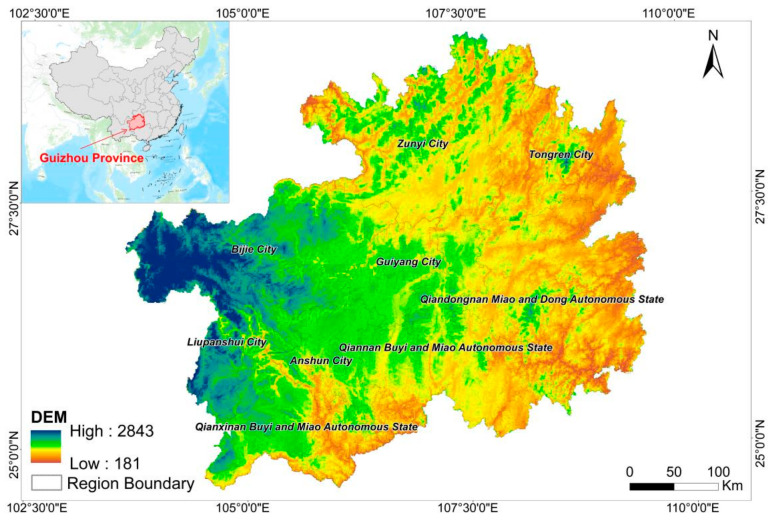
Overview map of the study area.

Guizhou Province (24°37′ N–29°13′ N, 103°36′ E–109°35′ E) is located in Southeast China (Figure 1), with a total area of 176,200 km^2^ [39]. It is adjacent to Hunan in the east, Guangxi in the south, Yunnan in the west, and Sichuan and Chongqing in the north. It has jurisdiction over 6 prefecture-level cities, 3 autonomous prefectures, and a total of 9 prefecture-level administrative regions. Guizhou is located in the Yunnan Guizhou Plateau, and the terrain is high in the west and low in the east. It inclined from the middle to the north, east, and south, with an average elevation of about 1100 m [39]. Guizhou has a warm and humid climate, belonging to the subtropical warm and humid monsoon climate zone, with diverse vegetation and numerous rivers [39]. The landform can be generally divided into three basic types: plateau mountains, hills, and basins. About 92.5% of the landform comprise mountains and hills. The soil area of Guizhou is 159,100 km^2^, accounting for 90.4% of the total land area of the province, and the zonality of the soil belongs to the red soil–yellow soil zone of the central subtropical evergreen broad-leaved forest [39]. For agricultural production, the amount of soil resources in Guizhou is obviously insufficient, and the soil available for agriculture, forestry, and animal husbandry accounts for only 83.7% of the total area of the province [39]. The development of karst landforms in Guizhou is very typical, with a wide range of karst distribution. The karst (exposed) area is 109,084 km^2^, accounting for 61.9% of the total land area of the province [39]. By 2020, the GDP of Guizhou was 17,826.56 million yuan and the permanent population was 38.56 million [40].

### 2.2. Material Sources

#### 2.2.1. Basic Geographic Data

The raster data of land use and the administrative boundary vector data of Guizhou Province were both from the Resource and Environmental Science and Data Center (https://www.resdc.cn/ (accessed on 10 July 2022)). The raster data of land-use monitoring by remote sensing were based on the spatial information of land-use change in 1 km grids obtained from remote sensing satellite data covering every 5a interval in China. Land use was divided into 6 primary types of farmland, forest land, grassland, water, residential land, and unused land, as well as 25 secondary types. The accuracy of discriminating farmland reached 99%; the accuracy of grassland, forest land, and construction land reached 98%; and the comprehensive accuracy reached more than 95%, which met the data accuracy requirements for the study. The terrestrial ecosystem data were based on the remote sensing detection data of land use in Guizhou Province, and the classification system of the terrestrial ecosystem in Guizhou Province was constructed by referring to the identification and research of each ecosystem type by the Resource and Environment Science and Data Center (Table 1).

#### 2.2.2. Socio-Economic and Environmental Data

The social and economic development data came from the 2011 and 2021 statistical yearbooks on the official websites of the people’s governments of cities and autonomous prefectures in Guizhou Province, the 2020 annual report of the Natural Resources and Planning Bureau on government information disclosure, and the data of the Statistics Bureau. The environmental data came from the Guizhou Provincial Water and Soil Conservation Bulletin (2016–2020) and the Guizhou Provincial Second Rocky Desertification Monitoring Results Bulletin (2020).

#### 2.2.3. Land-Use Driving Data

The data of land use and its driving factors included terrestrial ecosystem data, socio-economic data, and climatic and environmental data. The terrestrial ecosystem data were obtained after the classification of land-use remote sensing monitoring data in Guizhou Province in 2010 and 2020. The socio-economic data and climatic environment data were raster data with a resolution of 1 km × 1 km. The specific sources of data are shown in Table 2.

### 2.3. Research Methods

#### 2.3.1. Carbon Storage Assessment with the InVEST Model

InVEST (Integrated Valuation of Ecosystem Services and Tradeoffs) is a modeling system developed by the Natural Capital Project Team of the United States to assess ecosystem services and their economic value, as well as to support ecosystem management and decision making [41]. At present, the InVEST model has been widely used in spatial planning, ecological compensation, risk management, and climate change adaptation in many countries and regions [34]. Based on land use, land cover, and 4 carbon density pools (above-ground biomass, below-ground biomass, soils, and dead organic matter), the carbon storage in a current landscape or over a time period can be estimated with the InVEST model. Extremely unstable carbon in aboveground carbon pools (e.g., grasslands and short-period crops) are not considered because these carbon sources are relatively scarce, rapidly renewing, or very stable. In this study, the carbon storage for various land-use types was calculated using the following equation.

C*_i__*_tot_ = C*_i__*_above_ + C*_i__*_below_ + C*_i__*_soil_ + C*_i__*_dead_ (1)

where *i* is land-use type; C*_i__*_tot_ is the total carbon density of the land-use type *i*; C*_i__*_above_ is the aboveground biomass, including the carbon density of all living plant materials above the soil (such as bark, trunk, branches, and leaves); C*_i__*_below_ is the carbon density of the underground biomass, including the carbon density of the living roots of plants; C*_i__*_soil_ is the soil carbon density, which is usually the organic carbon of mineral soil but also includes organic soil; and C*_i__*_dead_ is the dead organic carbon density, including fallen leaves or dead trees. The carbon density database of the Guizhou terrestrial ecosystem was constructed after referring to the existing research of some scholars in the same research area (Table 3).

#### 2.3.2. Carbon Storage Prediction with the PLUS Model

**Figure 2:**
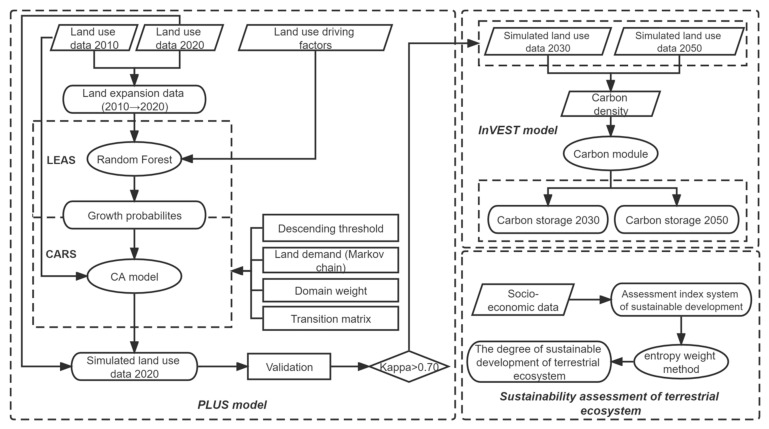
Flow chart of this study.

PLUS (Patch-generating Land Use Simulation) can explore the causal factors of various types of land-use changes and simulate land-use changes at the patch level [42]. The model includes two modules: Land Expansion Analysis Strategy (LEAS) and CA based on multiple random seeds (CARS). The LEAS module is able to extract and sample the construction expansion part of land-use change data in two periods, as well as to use the random forest algorithm to obtain the development probability and contribution of driving factors for each land-use type [46]. The CARS module combines random seed generation and a threshold-decreasing mechanism to simulate patches of automatic generation under the constraint of development probability [46]. A specific method flow chart is shown in Figure 2. First, based on the PLUS model, the LEAS module was used to analyze land-use expansion from 2010 to 2020. Then, a Markov Chain was used to calculate the demand for each land-use type in 2030 and 2050. Finally, the CARS module was used to simulate and forecast land-use changes in 2030 and 2050. The land-use simulation data were combined with the carbon density database to obtain the carbon storage projection results of Guizhou Province in 2030 and 2050.

This study only discussed land-use changes under a natural development scenario. Referring to some previous research [32,33,34,46], the various parameter settings in each module of the LEAS and CARS were as follows after multiple debugging sessions.

1. Random forest parameter: Random sampling was selected as the sampling method. The default sampling rate was 0.01. The number of mTry and decision trees was 9 and 20, respectively. The number of parallel threads was 6. The number of mTry generally did not exceed the number of driving factors, and two-thirds of the number of driving factors were selected here; the high number of parallel threads could improve the efficiency of operations [46].
2. CARS simulation parameter: The neighborhood size was 3 by default. The patch generation was the attenuation coefficient of the decreasing threshold, and the range was 0–1. The higher the value, the less likely it was to convert the type of land use [46]. The patch generation value was 0.5. The expansion coefficient was the probability of random patch seeds, and the range was 0–1. The higher the value, the easier it was to produce new patches [46]. The expansion coefficient value was 0.5, and the percentage of seeds was 0.05 by default. These settings allow for the shifting of land-use types in line with current socio-economic developments.
3. Transition matrix: In the transition matrix, 0 meant that one land-use type could not be converted into another land-use type, and 1 meant that it could be converted. Since water is a restrictive factor, water was not allowed to be converted into other land types. Construction land is not easy to change, so it was also set to not be converted into other land types. The amount of future land-use demand was calculated with a Markov Chain.
4. Domain weight: The domain weight is the expansion capacity of the land-use type, and the larger the weight, the larger the expansion capacity of the land type [32]. In this paper, the domain weight was determined by the proportion of expansion area of land-use type based on the LEAS. The weight of the farmland ecosystem was 0.1470, that of the forest ecosystem was 0.1841, that of the grassland ecosystem was 0.0774, that of the aquatic ecosystem was 0.0055, that of the settlement ecosystem was 0.0117, and that of the other ecosystem was 0.0001.

#### 2.3.3. Establishment of Assessment Index System for Sustainable Development of Terrestrial Ecosystems

Based on previous research results [47,48,49,50,51] and SDGs, combined with the characteristics of the terrestrial ecosystem in Guizhou Province, an assessment index system for the sustainable development of the terrestrial ecosystem was established. The evaluation index system of the sustainable development of the Guizhou terrestrial ecosystem is shown in Table 4.

The weight of each indicator was calculated using the entropy weight method. To eliminate the effects of numerical and unit differences, the data needed to be standardized. A positive indicator indicates that the larger the value of the indicator, the better the impact on the index layer. On the contrary, a negative indicator indicates that the larger the value of the indicator, the worse the impact on the index layer. The standardized formula is as follows:

(2) $$S_{ij}=\frac{P_{ij}-P_{\max}}{P_{\min}-P_{\max}}\;(\mathrm{positive}\;\mathrm{indicators})\;S_{ij}=\frac{P_{ij}-P_{\min}}{P_{\max}-P_{\min}}\;(\mathrm{negative}\;\mathrm{indicators})$$

where *S_ij_* is the standardized value, *P_ij_* is the original value of the *i*th item under the *j*th indicator, *P*_min_ is the minimum of all original values of the *i*th item, and *P*_max_ is the maximum of all original values of the *i*th item.

(3) $$S_{j}=-k\sum_{i=1}^{n}R_{ij}\cdot \ln R_{ij},\;k=\frac{1}{\ln n}$$

(4) $$W_{j}=\frac{\left(1-S_{j}\right)}{\sum_{j=1}^{m}\left(1-S_{j}\right)}$$

(5) $$E=\sum P_{ij}W_{j}$$

where *S_j_* is the entropy value of the *j*th indicator, *R_ij_* is the proportion of the value of the *i*th item under the *j*th indicator to the sum of all items, *n* is the number of items, *W_j_* is the weight, and *E* is the final calculated sustainable development value.

## 3. Results

### 3.1. Evolution of Terrestrial Ecosystem Carbon Storage in Guizhou Province

**Figure 3:**
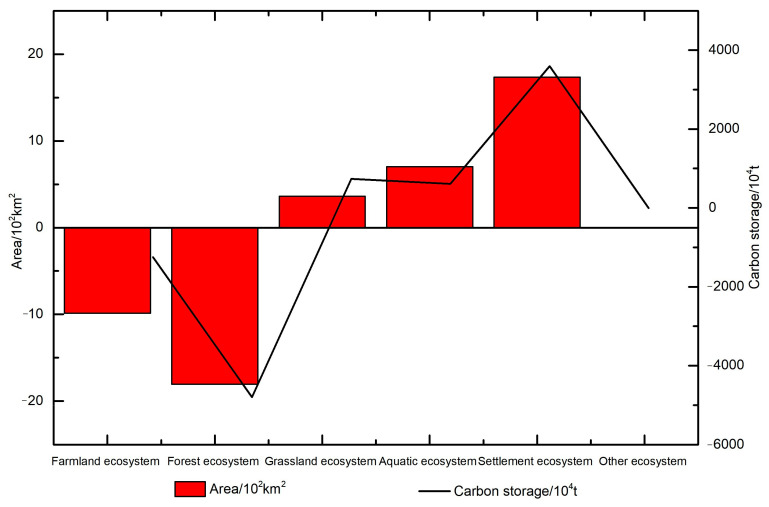
Area and carbon storage changes of the terrestrial ecosystem in Guizhou Province from 2010 to 2020.

From 2010 to 2020, the area of the grassland, aquatic, and settlement ecosystems in Guizhou Province increased by 363, 704, and 1736 km^2^, respectively, and the carbon storage increased by 608.75 × 10^4^, 736.20 × 10^4^, and 3595.60 × 10^4^ Mg, respectively (Table 5 and Figure 3). The area of the settlement ecosystem rapidly increased in the past 10 years, with the largest increase of 0.99%. The percentage change in the carbon storage of the grassland and aquatic ecosystems was 0.24% and 0.40%, respectively. The area of the farmland and forest ecosystems shrunk by 986 (0.56%) and 1806 km^2^ (1.03%), respectively. The area of the forest ecosystem decreased the most. Due to the reduction in the area, the carbon storage of the farmland and forest ecosystems also decreased by 1243.84 × 10^4^ (0.28%) and 4797.82 × 10^4^ Mg (1.08%), respectively. The other ecosystem only showed slight changes in area and carbon storage.

In general, the forest ecosystem accounted for the highest proportion of terrestrial ecosystems in Guizhou Province. The area of the forest ecosystem accounted for 52.83% by 2020, and its proportion of carbon storage was 65.50% of the whole province. The second highest proportion was in the farmland ecosystem, accounting for 27.42% of the provincial area and 16.15% of the carbon storage. It is worth mentioning that the area of the grassland ecosystem was only 17.71% but its proportion of carbon storage was the second highest, accounting for 16.76%. The forest and grassland ecosystems together accounted for 70.54% of the total area, providing 82.27% of the province’s carbon storage.

**Figure 4:**
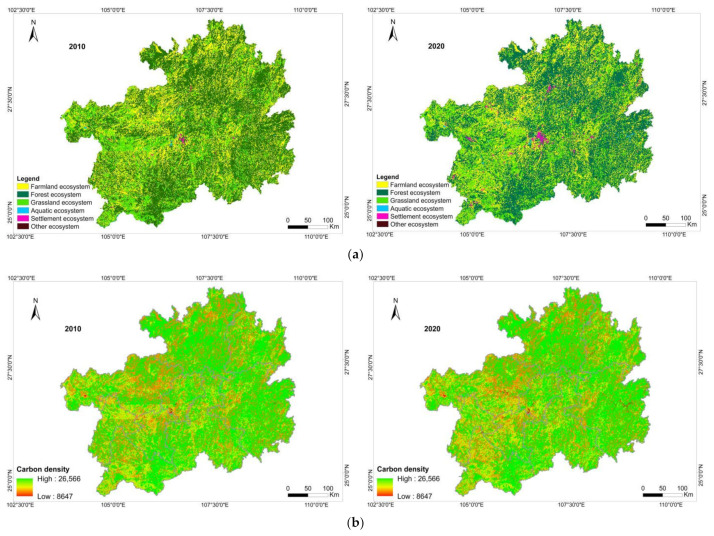
(**a**) Spatial distribution of the terrestrial ecosystem in 2010 and 2020; (**b**) spatial distribution of carbon storage in 2010 and 2020.

**Figure 5:**
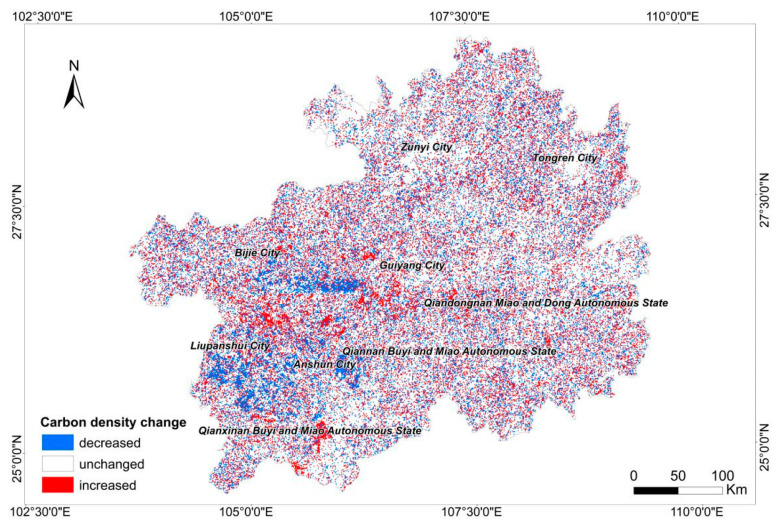
Carbon storage change map of the Guizhou terrestrial ecosystem from 2010 to 2020.

From the perspective of the spatial distribution of carbon storage (Figure 4), the regions with a low carbon density were mainly located in Bijie, Liupanshui, Anshun and Qianxinan. The carbon density in the north of Zunyi and the west of Tongren and Qiandongnan was also relatively low. The areas with low carbon density values were mainly the settlement and water ecosystems because the carbon density values of these two types of ecosystems were lower than those of the other terrestrial ecosystems. We used the grid calculator in ArcMap to obtain the spatial changes in the terrestrial ecosystem in the last 10 years and then calculated the corresponding carbon density value after the changes in the terrestrial ecosystem to obtain a carbon storage change map of the terrestrial ecosystem in Guizhou Province (Figure 5). Different degrees of transformation have occurred among various types of terrestrial ecosystems in various cities of Guizhou Province, most notably in the farmland, forest, and grassland ecosystems. From 2010 to 2020, 1649 km^2^ and 614 km^2^ of the farmland ecosystem were transferred to the forest ecosystem and the grassland ecosystem, respectively. A total of 1677 km^2^ and 677 km^2^ of the forest ecosystem were transferred to the farmland ecosystem and the grassland ecosystem, respectively. A total of 599 km^2^ and 608 km^2^ of the grassland ecosystem were converted into the farmland ecosystem and the forest ecosystem, respectively. Areas showing a significant decrease in carbon storage were mainly located in Bijie, Anshun, Liupanshui and Qianxinan, and a comparison with the transition of terrestrial ecosystems showed that the decreases in carbon storage were mainly caused by the transformation of the forest ecosystem to the grassland ecosystem and that the increases in carbon storage were caused by the conversion of the grassland ecosystem into the forest or farmland ecosystem.

### 3.2. Prediction of Carbon Storage of Terrestrial Ecosystem in Guizhou

**Figure 6:**
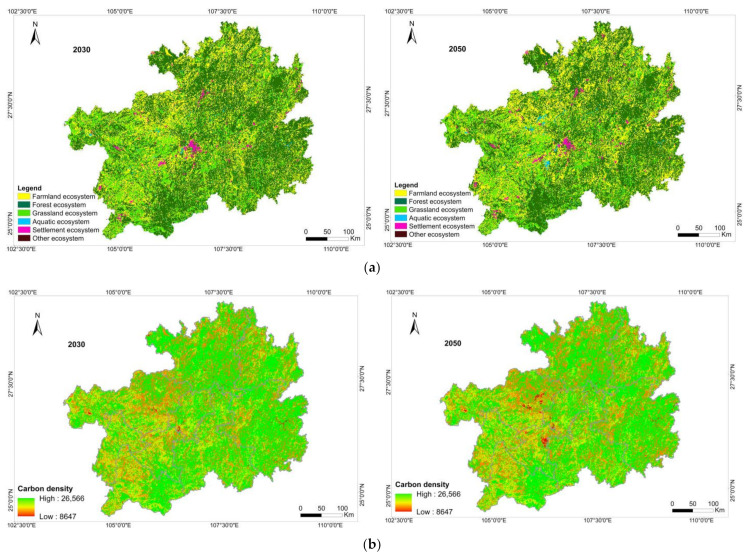
(**a**) Spatial distribution of the terrestrial ecosystem in 2030 and 2050; (**b**) spatial distribution of carbon storage in 2030 and 2050.

Based on the PLUS model and 13 driving factors, the land-use situations of Guizhou Province in 2030 and 2050 were established. The accuracy test showed that the Kappa coefficient was 0.76 and the total accuracy was 0.83. The accuracy test result was feasible and showed that the model could be used for simulation. By combining the carbon density data and simulated land-use data, the quantitative structure (Table 6) and spatial distribution (Figure 6) of the Guizhou terrestrial ecosystem’s carbon storage in 2030 and 2050 were simulated and predicted.

Compared with 2020, by 2030, the carbon storage will be 373,187.19 × 10^4^ Mg, with a decrease of 4091.43 × 10^4^ Mg. Among studied areas, the area of the farmland ecosystem will most rapidly decrease, by 1246 km^2^, and the carbon storage will decrease by 1571.83 × 10^4^ Mg. The carbon storage of the forest and grassland ecosystems will decrease by 1498.32 × 10^4^ Mg and 1397.36 × 10^4^ Mg, respectively. The area of the settlement ecosystem will increase by 238 km^2^, and the carbon storage will increase by 492.95 × 10^4^ Mg.

By 2050, the carbon storage of Guizhou Province is expected to reach 369,353.94 × 10^4^ Mg, with a total reduction of 3833.25 × 10^4^ Mg compared with 2030. Among the studied areas, the carbon storage of the forest ecosystem will decrease by 6216.44 × 10^4^ Mg, and the carbon storage of the grassland ecosystem will decrease by 1512.96 × 10^4^ Mg. The carbon storage of the farmland ecosystem will substantially increase by 3512.02 × 10^4^ Mg, and the carbon storage of the aquatic and settlement ecosystems will increase by 173.80 × 10^4^ Mg and 211.26 × 10^4^ Mg, respectively. The prediction results showed that by 2050, the area of the farmland ecosystem in Guizhou Province will increase by 2784 km^2^ compared with 2030, and the area of the forest ecosystem will decrease by 2340 km^2^. The area of the grassland ecosystem will increase by 746 km^2^, the area of the aquatic ecosystem will increase by 201 km^2^, and the area of the settlement ecosystem will increase by 102 km^2^. The main change in this stage is the conversion of the farmland and forest ecosystems. This will be manifested in a large-scale transformation of the forest ecosystem into the farmland ecosystem, leading to a reduction in the carbon storage of the forest ecosystem and an increase in the carbon storage of the farmland ecosystem.

Compared with 2020, the spatial distribution of carbon storage in the Guizhou terrestrial ecosystem will not change much in 2030. The areas with a low carbon density will still be located in Bijie, Liupanshui, Anshun and Qianxinan. Compared with 2030, the regions with significantly reduced carbon storage in 2050 will be located in the central part of Bijie and the eastern part of Anshun. The significant decrease will be due to the fact that the terrestrial ecosystems in these two areas will mainly be converted into an aquatic ecosystem, and the carbon density of aquatic ecosystems is low, thus resulting in a significant decline in carbon storage.

### 3.3. Assessment of Sustainable Development of Terrestrial Ecosystem in Guizhou Province

The sustainable development scores of the terrestrial ecosystem in Guizhou in 2020 are shown in Table 7. Among them, the sustainable development score of the terrestrial ecosystem in Zunyi was the highest at 0.6255 and the sustainable development score of the terrestrial ecosystem in Anshun City was the lowest at 0.3236. The average sustainable development score of the terrestrial ecosystem in Guizhou Province was found to be 0.4300. The sustainable development capacity of the land ecosystems in Zunyi, Guiyang, and Tongren was higher than the average level in Guizhou Province, and the sustainable development capacity of the terrestrial ecosystem in other cities (states) was lower than the average level. In terms of the weights of the selected SDGs, the sum of the weights of the four indicators of agricultural output value, urbanization rate, cultivated area, and grain output was found to reach 0.5110. Therefore, in the evaluation process, the higher the value of these indicators, the higher the score of the terrestrial ecosystem sustainability assessment. Among them, the agricultural output value had the highest weight, followed by the urbanization rate, cultivated land area, and grain output. Liupanshui and Anshun, with the lowest sustainability scores, had low agricultural output, grain output, and urbanization rates. The terrestrial ecosystem of Bijie and Qiandongnan had a low level of sustainable development. Although Bijie had the largest cultivated land area, its grain production was not high and its urbanization rate level was the lowest. The soil erosion area, rocky desertification area, and industrial waste gas SO_2_ emissions were the highest in Bijie. Zunyi had the highest degree of sustainable terrestrial ecosystem development due to its highest grain output and second highest agricultural output value and urbanization rate.

**Figure 7:**
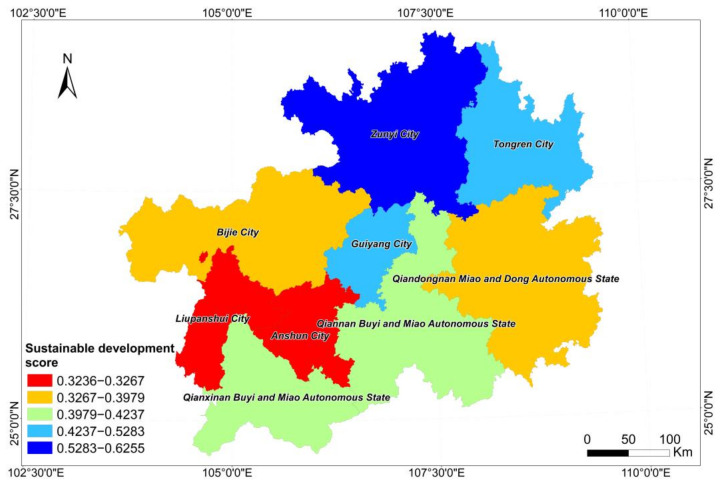
Assessment results of the sustainable development of the terrestrial ecosystem.

The sustainability scores of each city (state) were visualized using the quantile method to obtain Figure 7. It can be seen that the sustainable development ability was high in the north, low in the south, high in the east, and low in the west, which was related to the topography of Guizhou Province. Since the topography of Guizhou Province is high in the west and low in the east (sloping from the center to the north, east, and south), the areas with a higher topography were not conducive to urban construction and agricultural development. Karst areas are widely distributed in Guizhou Province, which has a fragile ecological environment and is prone to ecological and environmental problems in the process of development and utilization. According to the spatial distribution of the terrestrial ecosystem, the settlement ecosystem and the farmland ecosystem were also found to be concentrated in the low terrain areas.

## 4. Discussion

### 4.1. Reasons for the Carbon Storage Change in Terrestrial Ecosystem in Guizhou Province

The carbon storage has shown a decreasing trend in the past decade, mainly due to the reduction in the area of the farmland and forest ecosystems. In karst areas, changes in land use and land cover have significantly affected the carbon storage change in the terrestrial ecosystem, especially the forest ecosystem. After the conversion of cultivated and forest land into other lands, the carbon storage of the farmland and forest ecosystems decreased. The vegetation carbon storage values of the karst landform were higher than those of the non-karst landform [38]. Forest and cultivated land contribute the most carbon storage, and the reduction in their area led to the loss of carbon storage. This result aligns with Chen’s research [24]. The area of the farmland ecosystem was reduced while the area of the grassland ecosystem was slightly increased during this period. However, in this stage, construction land as also expanding, occupying the ecological service land to a certain extent and causing the loss of carbon storage. The forest ecosystem was shown to play an important role in the carbon storage of karst areas. A karst landscape can store an amount of organic carbon derived from forest ecosystem biomass [53]. The area of the forest ecosystem was reduced, so the carbon storage of the terrestrial ecosystem in Guizhou showed a decreasing tendency.

Land-use change has an important impact on soil erosion in karst areas, and reductions in vegetation cover exacerbate soil erosion [54]. The Guizhou Provincial Water and Soil Conservation Bulletin (2016–2020) and the study of Green SM. et al. [55] also showed that soil erosion in Guizhou Province mainly occurs in steep-slope cultivated land, barren hills and slopes, low-cover forest land, and areas of concentrated production and construction activity. Soil erosion in Guizhou Province showed a spatial pattern of a gradual reduction from northwest to southeast. Soil erosion and rocky desertification are important factors that restrict the development of karst areas. Rocky desertification prevents vegetation from growing and reduces carbon storage in the terrestrial ecosystem, which also limits the sustainable development of regional agriculture.

For non-karst areas, the farmland ecosystem showed the largest amount of carbon sequestration because the area of the forest ecosystem is too small [56,57]. The loss of carbon storage in non-karst areas, especially in the rapidly developing urbanized areas, is mainly due to urban land expansion [58]. The results of Sadat et al. also found that urban development and built lands had the considerable effect of reducing carbon storage in a nearby town with broad-leaved forests [59]. 

### 4.2. Suggestions for Sustainable Development of Terrestrial Ecosystem in Guizhou

According to the simulation results of the carbon storage of the terrestrial ecosystem in Guizhou Province, the carbon storage reduction areas in the next 30 years will still mainly be located in Bijie, Anshun, Liupanshui and Qianxinan. By 2050, the carbon storage of the terrestrial ecosystem in these cities (states) will continue to decline. The assessment results of the sustainable development status of the terrestrial ecosystem indicated that the sustainability of these cities was not high due to the influence of natural background conditions.

For these cities with a low degree of sustainable development, Guizhou Province first needs to firmly guard the two bottom lines of development and ecological protection, as well as enact the Grain for Green Program and the ecological restoration project as long-term plans. On the one hand, long-term farming on steep hillsides with a thin soil layer can exacerbate soil erosion and rocky desertification [60]. On the other hand, soil organic carbon represents a major carbon pool in terrestrial ecosystems [61]. Serious soil erosion in karst areas leads to the loss of soil organic matter and a decrease in carbon storage [62]. The Grain for Green Program helps to increase the vegetation cover and carbon storage in karst regions [63]. Although Guizhou Province has been promoting the implementation of the new round of the Grain for Green Program since 2014 and has achieved some goals, sustainable development still faces a huge challenge. With agricultural structure adjustment as the main line and the development of grass-fed animal husbandry as the focus, Guizhou Province should promote the sustainable development of grass-fed animal husbandry and realize the coordinated development of ecological benefits, economic benefits, and social benefits. Secondly, it is necessary to focus on ecological environmental protection. Finally, the government should accelerate the development of regional urbanization and strive to narrow the gap between urban and rural areas.

For cities with a high degree of sustainable development at present, it is important to continue to exploit the advantageous industries of each city. The karst landscape in Guizhou Province is widely distributed, and it is important to develop the tourism economy based on natural and human resources. Tourism is both a smokeless industry and a sustainable development industry. At the same time, we should strengthen regional cooperation and exchange to promote common development. Zunyi, with its convenient transportation and superior geographical location, can make full use of this location advantage and take the initiative to carry out cooperation and exchange with Guiyang, Bijie, Tongren, and Anshun to achieve resource sharing and complementary advantages.

### 4.3. Uncertainty Analysis

The InVEST model can quickly and efficiently calculate the carbon storage and distribution of each land-use type. However, because it assumes that the carbon density of each type does not change over time and with regional changes, its simulated values will have some differences with actual carbon storage values. In this study, based on the reference of previous carbon density data, we tried to select data that were most similar to the physical geographic profile and socio-economic development of this study area, as well as to reduce the error in carbon density values to some extent.

When using the PLUS model for terrestrial ecosystem simulation and prediction, the simulation accuracy is affected by the parameter settings, data accuracy, and other factors. In this study, only the distribution of carbon storage in 2030 and 2050 under the current socio-economic and natural development scenarios was assumed and no multi-scenario simulations were conducted. Therefore, the simulated conditions did not fully represent the future development of the terrestrial ecosystems in Guizhou Province.

Since the current indicator system for assessing the sustainable development of terrestrial ecosystems is not comprehensive and complete, there were differences in the assessment process that may have affected the assessment results to a certain extent.

### 4.4. Future Improvement Direction

For future carbon storage calculations, more carbon density data from field sampling can be used. A gray prediction model can be used to predict changes in carbon density at further points in time, and then the InVEST model can be used for calculation that can improve the accuracy of carbon storage estimation to a certain extent.

For carbon storage simulations, the future distribution of carbon storage in a terrestrial ecosystem under ecological protection scenarios can be simulated and predicted. Yang et al. found that carbon storage under ecological protection scenarios can be improved to a certain extent and that the loss of carbon storage can be reduced [64]. Therefore, simulating the carbon storage in different scenarios can help the government formulate more scientific land-use planning and sustainable regional development policies.

For sustainable development assessment, the indicators of sustainable development need to be further optimized and expanded. In addition, geographical data can be used for regional terrestrial ecosystem sustainable development assessment, which can geographically visualize sustainable development and will be more targeted to evaluate the sustainable development of terrestrial ecosystems and to propose improvement measures.

## 5. Conclusions

In this study, the evolution characteristics, simulation results, and sustainable development status of terrestrial ecosystem carbon storage in Guizhou Province were evaluated and analyzed in detail, and the main conclusions are as follows:

1. The carbon storage of the terrestrial ecosystem in Guizhou Province decreased by 1106.68 × 10^4^ Mg in the past 10 years. The carbon storage of the forest and farmland ecosystems decreased, while those of the grassland and settlement ecosystems increased. The areas with a significant decrease in carbon storage were mainly located in the western part of Guizhou Province.
2. Compared with 2020, by 2030, the carbon storage of Guizhou’s terrestrial ecosystem will decrease by 4091.43 × 10^4^ Mg, mainly due to the continuous reduction in the area of the farmland, forest, and grassland ecosystems. Compared with 2030, the carbon storage of the terrestrial ecosystem will still be decreasing by 2050, with a total decrease of 3833.25 × 10^4^ Mg.
3. The sustainable development state of the terrestrial ecosystem in Guizhou Province was found to be in high in the north, low in the south, high in the east, and low in the west. In the sustainable development evaluation system, the evaluation index related to the farmland ecosystem had higher weights, so the sustainable development status of the farmland ecosystem had a greater impact on the sustainable development status of terrestrial ecosystems in cities (states). Cities located in mountainous areas with high terrain, such as Bijie, Anshun, and Liupanshui, were found to have a less optimistic sustainable development status. In contrast, cities such as Zunyi, Guiyang, and Tongren, where natural conditions are more suitable for agricultural development and urban expansion, were found to have a higher degree of sustainable development. In regions with a low level of sustainable development, rates of soil and water conservation are low and the area of rocky desertification is large. Soil erosion reduces soil organic matter, and rocky desertification lowers vegetation cover. These are important factors in the carbon storage of the karst areas of a terrestrial ecosystem. To increase regional carbon storage and maintain the sustainable development of karst areas in terrestrial ecosystems, more attention needs to be paid to the problems caused by rocky desertification and soil erosion. The Grain for Green Program and ecological restoration can solve these ecological and environmental problems in Guizhou.

## Figures and Tables

**Table 1:** Classification system of the Guizhou terrestrial ecosystem.

First Level Code	First Level Type	Second Level Code	Second Level Type
1	Farmland ecosystem	-	-
		11	Paddy field
		12	Dry land
2	Forest ecosystem	-	-
		21	Woodland
		22	Shrubwood
		23	Open woodland
		24	Other forest land
3	Grassland ecosystem	-	-
		31	High-coverage grassland
		32	Medium-coverage grassland
		33	Low-coverage grassland
4	Aquatic ecosystem	-	-
		41	Rivers and canals
		42	Lakes
		43	Reservoir ponds
		44	Mudflats
5	Settlement ecosystem	-	-
		51	Urban land
		52	Rural settlement
		53	Other construction land
6	Other ecosystem	-	-
		61	Bare land
		62	Bare rocky terrain

**Table 2:** Types and sources of land-use driving data.

Types	Data	Description
Terrestrial ecosystem data	Remote sensing monitoring data of land use in Guizhou Province in 2010	1—Farmland ecosystem; 2—Forest ecosystem; 3—Grassland ecosystem; 4—Water ecosystem; 5—Settlement ecosystem; 6—Other ecosystem
	Remote sensing monitoring data of land use in Guizhou Province in 2020	
Socio economic data	Spatial distribution of GDP in 1 km grid dataset	Resource and Environmental Science and Data Center (https://www.resdc.cn/ (accessed on 10 July 2022))
	Spatial distribution of population in 1 km grid dataset	
	Distance to urban first-class road	Open Street Map (https://www.openstreetmap.org/ (accessed on 5 July 2022)), calculated by Euclidean distance, with a resolution of 1 km × 1 km
	Distance to urban secondary road	
	Distance to urban third-class road	
	Distance to railway	
	Distance to provincial road	
	Distance to national highway	
	Distance to expressway	
Climatic and environmental data	Annual average temperature	Resource and Environmental Science and Data Center (https://www.resdc.cn/ (accessed on 10 July 2022))
	Annual average precipitation	
	Altitude	
	Slope	

**Table 3:** Carbon density of the Guizhou terrestrial ecosystem (Mg/hm^2^).

Types	C*_i_*__above_	C*_i_*__below_	C*_i_*__soil_	C*_i_*__dead_	C*_i_*__tot_	Sources
Farmland ecosystem	13.05	7.30	103.48	2.32	126.15	[24]
Forest ecosystem	20.36	67.50	170.00	7.80	265.66	[42,43,44]
Grassland ecosystem	0.82	0.87	199.84	1.28	202.81	[24,43,45]
Aquatic ecosystem	1.02	1.34	83.63	0.48	86.47	[24]
Settlement ecosystem	0.07	1.63	205.07	0.35	207.12	[24,45]
Other ecosystem	0.74	0.93	90.90	0.36	92.93	[24]

**Table 4:** The assessment index system for the sustainable development of a terrestrial ecosystem.

Target Layer	Index Layer	Interpretation	Unit	Corresponding SDGs	Indicator Properties	Max	Min	Std.	Weight
Sustainable development capacity of the Guizhou terrestrial ecosystem	Cultivated land area	Describes the sustainability of a farmland ecosystem.	hm^2^	15.1/2.1	+	1221.08	286.51	307.35	0.1158
	Grain output	Refers to the total output in the whole country including grains produced by state farms, collective units, and rural households, as well as farms affiliated with industrial and mining enterprises and other production units; used to measure food security.	10,000 t	2.1	+	234.7	37.64	72.03	0.1039
	Proportion of secondary and tertiary industries in GDP	Measures the proportion of industry.	%	9.2	+	95.86	75.92	5.98	0.0771
	Agricultural output value	Refers to the total amount of all the products of agriculture, forestry, animal husbandry, and fisheries expressed in monetary terms for a certain period of time (usually one year); used to reflect the total scale and results of agricultural production.	10,000 yuan	2.3	+	5,484,093	1,905,948	1,384,602.63	0.1600
	GDP per capita	Measures the level of economic development.	10,000 yuan per person	8.1/8.2	+	7.22	2.93	1.30	0.0839
	Urbanization rate	Refers to the proportion of urban population in the total population; used to describe the quality and environment of the settlement ecosystem.	%	11.3	+	80.07	42.12	11.50	0.1314
	Rocky desertification area	Refers to the evolution of surface vegetation and soil in karst areas into rocky landscapes with almost no vegetation and soil [52]; used to measure land quality and land degradation.	hm^2^	15.3	−	49.68	10.98	12.17	0.0484
	Forest cover rate	Refers to the ratio of area of afforested land to total land area. It is a very important indicator that reflects the status of the abundance of forest resources and ecosystem balance.	%	15.1/15.2	+	68	55	3.99	0.0474
	Soil erosion area	Measures land quality and land degradation.	km^2^	6.6/13.1	−	9959.07	1799.56	2609.19	0.0491
	Decrease in rate of energy consumption per unit of GDP	Refers to the energy consumed by a country or region for each unit of GDP produced; used to describe improvements in energy efficiency.	%	7.3	+	4.05	−0.82	1.49	0.0427
	Industrial wastewater discharged	Refers to the volume of waste water discharged by industrial enterprises through all their outlets; used to describe the efficient disposal of waste.	10,000 t	12.4	−	7024.63	80	2456.10	0.0494
	Industrial waste gas SO_2_ emissions	Refers to the volume of sulfur dioxide emission from the fuel burning and production processes by enterprises; used to describe the efficient disposal of waste.	t	12.4	−	34,573.91	4077.82	9888.96	0.0472
	Consumption of chemical fertilizers in agriculture	Refers to the quantity of chemical fertilizers applied in agriculture in a year; used to describe the pollution of the farmland ecosystem.	t	Non-SDGs	−	181,584	47,935	45,889.84	0.0437

**Table 5:** Terrestrial ecosystem area and carbon storage in Guizhou Province from 2010 to 2020.

Types	2010		2020	
	Area/km^2^	Carbon Storage/10^4^ Mg	Area/km^2^	Carbon Storage/10^4^ Mg
Farmland ecosystem	49,275	62,160.41	48,289	60,916.57
Forest ecosystem	94,833	251,933.35	93,027	247,135.53
Grassland ecosystem	30,821	62,508.07	31,184	63,244.27
Aquatic ecosystem	485	419.38	1189	1028.13
Settlement ecosystem	642	1329.71	2378	4925.31
Other ecosystem	37	34.38	31	28.81
Sum	176,093	378,385.30	176,098	377,278.62

**Table 6:** Quantitative structure of the carbon storage of the terrestrial ecosystem in 2030 and 2050.

Types	2030			2050		
	Area/km^2^	Carbon Storage/10^4^ Mg	Carbon Storage Change/10^4^ Mg (2020–2030)	Area/km^2^	Carbon Storage/10^4^ Mg	Carbon Storage Change/10^4^ Mg (2030–2050)
Farmland ecosystem	47,043	59,344.74	−1571.83	49,827	62,856.76	3512.02
Forest ecosystem	92,463	245,637.21	−1498.32	9012	239,420.76	−6216.44
Grassland ecosystem	30,495	61,846.91	−1397.36	29,749	60,333.95	−1512.96
Aquatic ecosystem	1056	913.12	−115.01	1257	1086.93	173.80
Settlement ecosystem	2616	5418.26	492.95	2718	5629.52	211.26
Other ecosystem	29	26.95	−1.86	28	26.02	−0.93
Sum	173,702	373,187.19	−4091.43	173,702	369,353.94	−3833.25

**Table 7:** Sustainable development capacity of the terrestrial ecosystem in each city.

City (State)	Sustainable Development Capacity of Terrestrial Ecosystem
Guiyang	0.5283
Zunyi	0.6255
Liupanshui	0.3267
Anshun	0.3236
Bijie	0.3979
Tongren	0.4769
Qianxinan	0.4049
Qiandongnan	0.3626
Qiannan	0.4237
Guizhou Province	0.4300

## Data Availability

All the data in this study can be found here: Resource and Environmental Science and Data Center (https://www.resdc.cn/ (accessed on 10 July 2022)), OpenStreetMap (https://www.openstreetmap.org/ (accessed on 5 July 2022)), Website of the People’s Government of Guizhou Province (http://guizhou.gov.cn/ (accessed on 2 April 2022)), and Guizhou Provincial Bureau of Statistics (http://stjj.guizhou.gov.cn/ (accessed on 3 April 2022)).
